# Altered Spontaneous Activity in Anisometropic Amblyopia Subjects: Revealed by Resting-State fMRI

**DOI:** 10.1371/journal.pone.0043373

**Published:** 2012-08-24

**Authors:** Xiaoming Lin, Kun Ding, Yong Liu, Xiaohe Yan, Shaojie Song, Tianzi Jiang

**Affiliations:** 1 State Key Laboratory of Ophthalmology, Zhongshan Ophthalmic Center, Sun Yat-sen University, Guangzhou, China; 2 LIAMA Center for Computational Medicine, National Laboratory of Pattern Recognition, Institute of Automation, The Chinese Academy of Sciences, Beijing, China; 3 Key Laboratory for NeuroInformation of Ministry of Education, School of Life Science and Technology, University of Electronic Science and Technology of China, Chengdu, China; 4 The Queensland Brain Institute, The University of Queensland, Brisbane, Queensland, Australia; Banner Alzheimer's Institute, United States of America

## Abstract

Amblyopia, also known as lazy eye, usually occurs during early childhood and results in poor or blurred vision. Recent neuroimaging studies have found cortical structural/functional abnormalities in amblyopia. However, until now, it was still not known whether the spontaneous activity of the brain changes in amblyopia subjects. In the present study, regional homogeneity (ReHo), a measure of the homogeneity of functional magnetic resonance imaging signals, was used for the first time to investigate changes in resting-state local spontaneous brain activity in individuals with anisometropic amblyopia. Compared with age- and gender-matched subjects with normal vision, the anisometropic amblyopia subjects showed decreased ReHo of spontaneous brain activity in the right precuneus, the left medial prefrontal cortex, the left inferior frontal gyrus, and the left cerebellum, and increased ReHo of spontaneous brain activity was found in the bilateral conjunction area of the postcentral and precentral gyri, the left paracentral lobule, the left superior temporal gyrus, the left fusiform gyrus, the conjunction area of the right insula, putamen and the right middle occipital gyrus. The observed decreases in ReHo may reflect decreased visuo-motor processing ability, and the increases in ReHo in the somatosensory cortices, the motor areas and the auditory area may indicate compensatory plasticity in amblyopia.

## Introduction

Amblyopia, or “lazy eye”, is a disorder of the visual system characterized by visual deficiency in an eye that is otherwise physically normal or by a deficiency that is out of proportion with the structural abnormalities of the eye [1], [2], [3]. Amblyopia is also one of the most common causes of unilateral visual deficits in adulthood [4]. In recent years, extensive neuroimaging studies have been carried out to assess cortical structural/functional abnormalities in amblyopia. Several studies have detected morphologic changes, such as reduced gray matter, in the visual cortical regions of amblyopia subjects using a voxel-based morphometry (VBM) analyses [5], [6]. These findings may indicate developmental abnormalities of the visual cortex during the critical growth period. Additionally, the advent of fMRI has produced convergent evidence of cortical dysfunction in amblyopia [7], [8], [9]. Lv et al. [10] investigated the functional dysfunction of the visual cortex that accompanies morphological changes in amblyopia and found that functional deficits may be correlate with gray matter volume in some areas, especially the occipital lobe, indicating that visual dysfunction may potentially have structural substrates in human amblyopia. Other neuroimaging tools, such as positron emission tomography and magnetoencephalography, have also provided evidence that a number of cortical areas, including the primary and secondary visual areas, regions within the parieto-occipital cortex, and the ventral temporal cortex, show reduced levels of activation in amblyopia [11].

It is generally believed that the visual cortex is the principle site of vision deficits in the visual pathway in amblyopia [7], [8], [12]. Previous studies have also observed functional deficits and morphological changes at the level of the lateral geniculate nucleus (LGN) in anisometropic amblyopia [13], [14], [15]. For example, Miki et al. [15] found that activation of the LGN is significantly affected during monocular viewing with the affected eyes. Hess et al. [14] also demonstrated that functional deficits are observable at the thalamic level using high-field fMRI in a group of human adults with amblyopia. Barnes et al. [13] found that the LGN is structurally abnormal in humans with strabismic amblyopia. Additionally, some studies have suggested that extra-striate regions are also responsible for abnormalities in special visual function in amblyopia. Barnes et al. [8] measured fMRI activation between the fixing and fellow amblyopic eyes of ten individuals with strabismic amblyopia and found that stimuli that were well within the amblyopic passband produced reduced fMRI activation in visual areas V1, V2, and V3A. Simmers and colleagues' studies suggest that, in addition to striate cortical dysfunction, both ventral and dorsal extra-striate function are affected by amblyopia, and these effects are not simply consequences of striate dysfunction [16], [17], [18].

In contrast to task-based fMRI studies, resting-state fMRI requires neither stimulation nor response and reflects the spontaneous neuronal activity or the background neurophysiological processes of the human brain [19]. Using resting-state fMRI, Biswal et al. [20] found that spontaneous low-frequency fluctuations in blood oxygen level dependent (BOLD) signals were highly synchronous within the somato-motor system and concluded that these fluctuations were physiologically meaningful. Since then, there has been increased interest in resting-state fMRI, and this method has been used in healthy subjects to investigate brain activities in various functional systems (including motor, auditory, visual, language and limbic systems), and in neurological and psychiatric diseases (including multiple sclerosis, schizophrenia and major depression) [21], [22], [23].

Zang et al. [24] proposed Regional Homogeneity (ReHo) as an index for measuring activity patterns during resting states. ReHo is a rank order statistic measure that evaluates the similarity between the time series of a given voxel and its nearest neighbors. The ReHo measure can rapidly map the level of regional activity across the whole brain of an individual [25]. Based on the hypothesis that brain activity works in clusters rather than in a single voxel, the ReHo index is robust to differences in the magnitude of the BOLD response. Regions with higher ReHo values may indicate that these brain areas have greater similarities in activity with their neighbors. In previous studies, this index has been successfully used to study a variety of neurological and psychiatric diseases (such as schizophrenia, Parkinson's disease, blindness, Alzheimer's disease etc.) and has provided new disease-related findings [26], [27], [28], [29], [30], [31], [32], [33], [34]. Regarding activity patterns of the visual cortex, Liu et al. [33] reported that blind subjects have higher ReHo values in the visual cortex compared with subjects with normal vision; this finding may be explained by abnormal cortical development and/or experience dependent plasticity in the visual cortex. The studies mentioned above indicate that the analysis of ReHo during resting states may provide a new approach to explore functional abnormalities of spontaneous brain activity. Until now, the spontaneous brain activity patterns of amblyopia subjects were still unclear. Thus, we used ReHo to address the question of where and how brain activity patterns are impaired in amblyopia.

## Materials and Methods

### Subjects

The study was approved by the Ethics Committee of Zhong Shan Ophthalmic Center, Sun Yat-sen University and followed the tenets of the Declaration of Helsinki. Written informed consent was obtained from all participants enrolled in the study or their legal guardians, and all participants received detailed eye examinations that included assessments of visual acuity, intraocular pressure and refraction, slit lamp examination, ophthalmoscopy, binocular alignment, ocular motility, and random-dot butterfly stereograms. In total, fourteen amblyopic patients and twenty-two healthy individuals were enrolled in the study. Two participants (1 healthy volunteer, 1 patient with amblyopia) had excessive head motion during scanning (see data preprocessing) and were excluded, leaving twenty-one healthy volunteers and thirteen patients with amblyopia who were included in the analysis. All the subjects were right-handed and had no history of strabismus, other ocular diseases, surgery, neurological disorders, or brain abnormalities based on MRI scans. The volunteers had normal or corrected to normal visual acuity in both eyes. Detailed clinical data of the subjects are shown in Table 1.

**Table 1:** Demographic characteristics of the participants with anisometropic amblyopia.

Subject	Gender	Age (Y)	amblyopia eye	Acuity (20/feet)		Refraction	
				Right Left	Left	Right	Left
AN01	M	18	AE (L)	20/12.5	20/62.55	plano	+3.25DS/+2.25DC×80
AN02	M	22	AE (OU)	20/100	20/100	+10 DS/+0.5DC×180	+11.5DS/+0.25DC×80
AN03	F	18	AE (R)	20/200	20/12.5	+6.5DS/+0.5DC×90	plano
AN04	F	22	AE (R)	20/200	20/20	−19.5DS/−1DC×65	−6.0DS
AN05	F	18	AE (R)	20/80	20/32	+8.0DS/+0.75DC×120	+7.0DS/+1DC×60
AN06	F	20	AE (L)	20/20	20/200	−5.5DS	−20DS
AN07	F	17	AE (R)	20/50	20/20	+2.75DS/−5.50DC×175	+0.50DS/+0.50DC×90
AN08	F	18	AE (L)	20/20	20/400	−1.5DS/−0.25DC×85	−13.0DS/−2.0DC×165
AN09	M	17	AE (L)	20/20	20/32	−2.5DS/−0.75DC×175	−7.25DS/−0.50DC×180
AN10	M	24	AE (R)	20/50	20/32	+7.75DS	+6.0DS/+0.75DC×90
AN11	F	30	AE (L)	20/16	20/50	+0.50DS/+0.50DC×70	+8.0DS/+1.0DC×120
AN12	M	23	AE (L)	20/12.5	20/400	+5.0DS	+7.0DS
AN13	F	43	AE (L)	20/20	20/5020/20	−0.5DC×75	+3.0DS

### Data acquisition

MRI data were obtained using a 3.0 Tesla MR scanner (Trio Tim system; Siemens, Erlangen, Germany). Resting-state fMRI scans were performed with an echo planar imaging (EPI) sequence with the following scan parameters: repetition time = 2000 ms, echo time = 30 ms, flip angle = 90°, matrix = 64×64, field of view = 220×220 mm^2^, slice thickness = 3 mm and slice gap = 1 mm. Each brain volume comprised 32 axial slices, and each functional run contained 270 volumes. During the scans, all subjects were instructed to keep their eyes closed, relax and move as little as possible. Tight, but comfortable, foam padding was used to minimize head motion, and earplugs were used to reduce scanner noise.

The structural magnetization prepared rapid gradient-echo imaging (MP-RAGE) sequence was used to acquire structural T1-weighted images in a sagittal orientation. The parameters were as follows: repetition time = 2000 ms; echo time = 2.6 ms; flip angle = 9°; acquisition matrix = 512×448; field of view = 256×224 mm^2^. The scanning time was approximately 5 min, and a total of 192 images with slice thicknesses of 1 mm were obtained.

### Data preprocessing

All preprocessing procedures were carried out using Statistical Parametric Mapping software (SPM8, http://www.fil.ion.ucl.ac.uk/spm). To allow subjects to adapt to the scanning situation and to obtain magnetization equilibrium, the first 10 volumes of each functional time series were discarded. The remaining fMRI volumes were slice corrected and then realigned to the first volume. Subject were included if their head movement during fMRI scanning was less than 3 mm translation in any axis and less than 3° angular rotation in any axis. All realigned data were then spatially normalized to the standard Montreal Neurological Institute (MNI) EPI template and resampled to 2×2×2 mm cubic voxels. To further reduce the effects of confounding factors, six motion parameters, linear drift and the mean time series of all voxels within the white matter and the cerebrospinal fluid were removed from the data by linear regression. After that, a temporal filter (0.01–0.08 Hz) was applied to reduce the effect of low-frequency drift and high-frequency noise signals. Finally, the regressed images were smoothed with a 6 mm full width at half maximum (FWHM) to reduce spatial noise [33], [34], [35].

Structural MRI data were preprocessed in SPM8 using voxel-based morphometry (VBM) as implemented in the VBM8 toolbox with default parameters (http://dbm.neuro.uni-jena.de/vbm.html). Images were segmented into gray matter (GM), white matter and cerebrospinal fluid tissue classes. Next, the segmented GM images were bias-corrected and registered with a template image in MNI space [36]. Finally, the modulated GM volumes were resampled to 2 mm cubic voxel resolution and smoothed with a 6 mm FWHM 3D Gaussian kernel.

### ReHo measurement and statistical analysis

In the present study, we investigated alterations in the spontaneous activity in anisometropic amblyopia subjects using ReHo. ReHo is a fast method for mapping regional spontaneous activity across the whole brain [24]. We do not provide the definition of ReHo here because it has been used many times, and detailed definitions can be found in Zang et al. [24].

To reduce the effect of individual variability, we normalized the ReHo value of each voxel by dividing it by the mean ReHo of the whole brain for each subject [28], [33], [34]. That is, for each voxel,
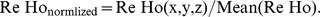



Next, a two-sample two-tailed *t*-test was performed to investigate the group differences in ReHo between the anisometropic amblyopia subjects and subjects with normal vision after regressing out the effects of age and gender. The statistical threshold for each voxel was set at *P*_alpha_<0.01, with a cluster size of at least 130 voxels, based on the results of a Monte Carlo simulation (http://afni.nimh.nih.gov/pub/dist/doc/manual/AlphaSim.pdf, AlphaSim with the following parameters: single voxel *P = 0.01*, FWHM = 6 mm, with AAL template in MirCroN software as a mask). Statistical comparisons of the mean fitted ReHo values between each pair of groups were performed using a two-sample two-tailed t-test at a threshold of P<0.05 (FDR corrected using groups times the number of significant brain regions). The VBM analyses were performed using the same statistical method.

### Relationship between ReHo and clinical variables

To determine whether the ReHo index varied with disease progression in the patients, correlation analyses between the ReHo index in the identified regions and each of the clinical variables (visual acuity of bilateral eyes) were performed. Further, to determine if structural alteration accompanies with the altered spontanous brain activity in the patients, we also evaluated the gray matter volume in the identified regions of the amblyopia subjects. Because these analyses were exploratory in nature, we used a statistical significance level of *P*<0.05 (uncorrected).

## Results

The demographic and psychological characteristics of the 13 patients with amblyopia (5 males, 8 females; mean age: 22.3±7.2 years) are summarized in Table 1. The 21 healthy volunteers individuals (8 males, 13 females; mean age: 23.5±2.1 years) were well matched with the amblyopia group in age (*P* = 0.47, two-sample two-tailed *t*-test) and gender (*P* = 0.98, Chi-squared test) distribution. Additionally, to further evaluate the influence of head motion on ReHo results, an extra evaluation of differences in movement parameters between amblyopia and subjects with normal vision was performed according the procedures described in Van Dijk et al. [37]. The mean motions of the subjects with anisometropic amblyopia and subjects with normal vision groups were 0.062±0.033 mm and 0.051±0.020 mm, respectively. No significant difference was found between the two groups (*P* = 0.219, two-sample, two-tailed t-test).

**Figure 1:**
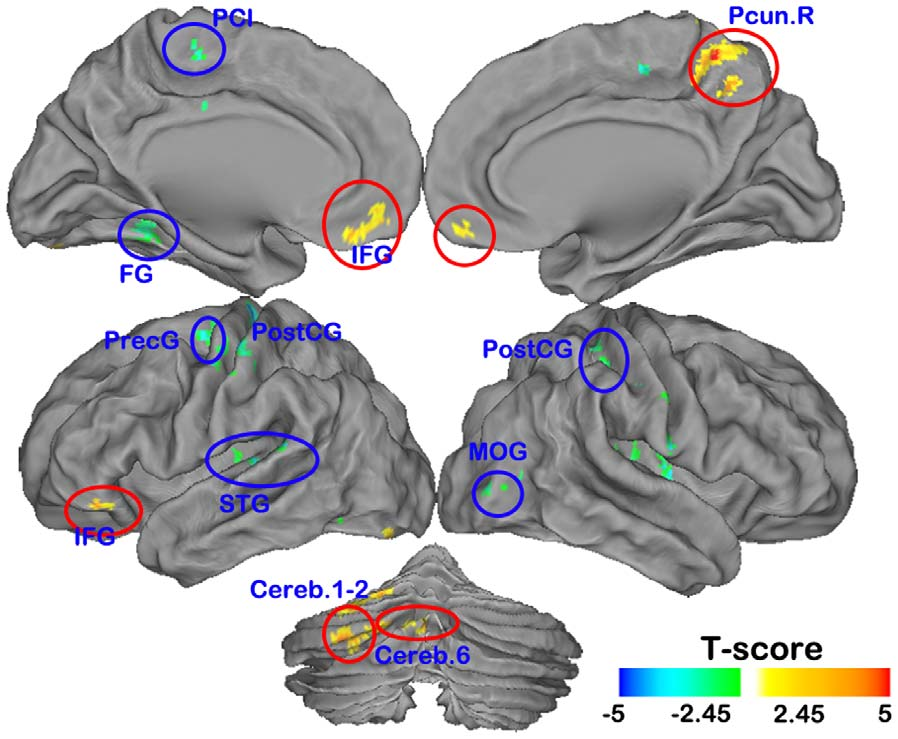
The anatomical distribution of the altered normalized ReHo in anisometropic amblyopia visualized by individuals Caret v5.61 software (*P<0.01*, 130 voxels, Alphasim corrected *P_alpha_* = 0.01). Abbreviations: Pcun = precuneus, IFG = inferior frontal gyrus, MPFC = media prefrontal cortex, Cereb = cerebellum; PostCG = postcentral gyrus, PretCG = precentral gyrus, PCL = paracentral lobule, STG = superior temporal gyrus, FG = fusiform gyrus, MOG = middle occipital gyrus.

**Figure 2:**
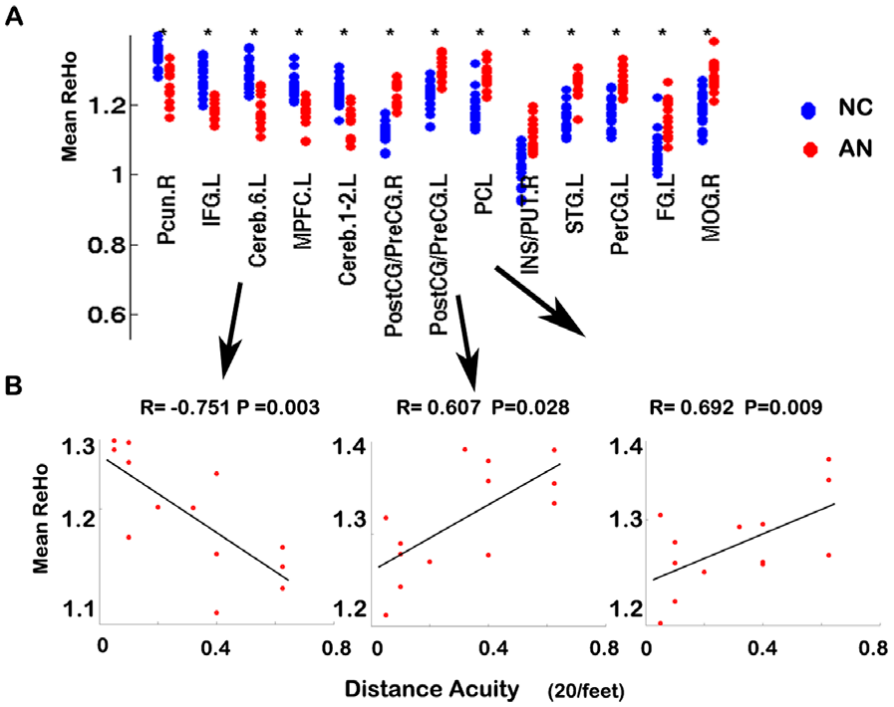
Plot of the normalized ReHo values in the brain areas in which ReHo values were significantly different between the subjects with normal vision and anisometropic amblyopia individuals(A). Correlation between the mean fitted ReHo indices and visual acuities in the patients with anisometropic amblyopia (P<0.05)(B). Abbreviations: same with Figure 1.

Compared with the subjects with normal vision, the anisometropic amblyopia individuals showed significantly decreased ReHo in brain regions including the right precuneus (Pcun), the left medial prefrontal cortex (MPFC), the left inferior frontal gyrus (IFG), and the left cerebellum (cerebellum area 1–2, 6) at the threshold in clusters larger than 130 voxels at P<0.01 (Alphasim corrected) (Figure 1 red, Figure 2 A and Table 2). We also found some brain areas [the bilateral conjunction area of the postcentral (PostGG) and precentral gyri (PreCG), the left paracentral lobule (PCL), the left superior temporal gyrus (STG), the left fusiform gyrus, the conjunction area of right insula and putamen and the right middle occipital gyrus (MOG)] with significantly increased ReHo at the threshold in clusters larger than 130 voxels at P<0.01 (Alphasim corrected) (Figure 1 blue, Figure 2 A and Table 3).

**Table 2:** Brain areas with decreased ReHo in anisometropic amblyopia individuals (*P<0.01*, 130 voxels, Alphasim corrected *P_alpha_* = 0.01).

Brain Region	Brodmann Region	Cluster Size	T-values	Z-scores	Coordinates in MNI (x, y, z)
Pcun.R	7	228	5.81	4.77	6 −52 56
			4.41	3.87	6 −56 44
			4.01	3.58	8 −54 64
IFG.L	45/47	209	5.65	4.67	−52 38 −4
			4.36	3.83	−56 26 6
			4.33	3.81	−40 34 −4
Cereb.6.L		132	4.93	4.22	−16 −76 −20
			3.93	3.52	−28 −82 −22
			3.22	2.98	−10 −68 −22
MPFC	10/11	191	4.74	4.10	−2 34 −16
			4.36	3.83	2 46 −10
			3.88	3.49	−10 42 −6
Cereb.1–2.L		184	4.70	4.06	−32 −86 −36
			4.25	3.75	−14 −90 −30
			4.05	3.61	−2 −84 −30

Abbreviations: Pcun = precuneus, IFG = inferior frontal gyrus, MPFC = media prefrontal cortex, Cereb = cerebellum.

L: left, R: right.

**Table 3:** Brain areas with increased ReHo in anisometropic amblyopia individuals (*P<0.01*, 130 voxels, Alphasim corrected *P_alpha_* = 0.01).

Brain Region	Brodmann Region	Cluster Size	T-values NC-AN	Z-scores	Coordinates in MNI (x, y, z)
PostCG/PreCG.R	3/4	522	−5.34	−4.48	24 −30 56
			−5.07	−4.31	46 −18 40
			−4.50	−3.93	58 −10 18
PostCG/PreCG.L	3/4	321	−5.26	−4.43	−42 −24 54
			−4.58	−3.99	−54 −18 46
			−4.46	−3.91	−48 −28 48
PCL	4/6	222	−5.02	−4.28	−20 −30 60
			−5.00	−4.27	−18 −30 68
			−4.34	−3.82	4 −24 52
INS/PUT.R		152	−4.99	−4.26	36 −10 6
			−4.32	−3.81	28 −12 6
			−3.74	−3.38	34 −14 18
STG.L		288	−4.87	−4.18	−42 −30 8
			−4.67	−4.05	−48 −28 20
			−4.26	−3.76	−32 −20 12
PerCG.L	6	224	−4.81	−4.14	−32 −16 68
			−4.56	−3.97	−36 −10 56
			−3.85	−3.47	−26 −14 60
FG.L	19/37	133	−4.65	−4.03	−38 −52 −8
			−3.96	−3.55	−28 −50 −14
			−3.37	−3.10	−40 −60 −12
MOG.R	18/19	157	−4.09	−3.64	28 −80 6
			−3.87	−3.48	34 −74 2
			−3.75	−3.39	44 −82 4

Abbreviations: PostCG = postcentral gyrus, PretCG = precentral gyrus, PCL = paracentral lobule, INS/PUT = insula and putamen, STG = superior temporal gyrus, FG = fusiform gyrus, MOG = middle occipital gyrus.

L: left, R: right.

As Figure 2 shows, ReHo in the left cerebellum (area 6), the left PostCG/PreCG and the PCL was significantly correlated with visual acuity in the anisometropic amblyopia individuals (P<0.05).

We did not find significant differences in gray matter volumes using the VBM method at our statistical threshold (P<0.01, AlphaSim corrected). Thus, our results demonstrate that gray matter volumes are not significantly altered in the identified regions in anisometropic amblyopia individuals (P<0.05, FDR corrected) (Figure S1).

## Discussion

To our knowledge, this is the first study to investigate spontaneous brain activity in anisometropic amblyopia. The present study demonstrated that spontaneous activity patterns of some brain regions in the anisometropic amblyopia individuals were altered when compared to subjects with normal vision.

### Decreased ReHo of spontaneous brain activity in amblyopia

The amblyopia subjects showed decreased ReHo of spontaneous brain activity in the right precuneus cortex (part of BA7) and the left cerebellum (cerebellum area 1–2, 6) (Figure 1, Table 2). The precuneus partially corresponds to the medial posterior parietal cortex, where previous studies have shown decreased glucose metabolism [38], [39] and loss of gray matter [6] in visually impaired subjects. Functionally, the medial posterior parietal cortex area is believed to play a role in visuo-motor coordination (e.g., in reaching to grasp an object) [39], [40], [41]. Similarly, the cerebellum, which functionally interacts with the frontal eye fields [42], [43], [44], [45], is also involved in the control of eye movements [46], [47], [48], [49], [50]. Neural cells in crus 1 and 2 are related to eye movements in monkeys during a visually guided eye movement tracking task [51]. In humans, the lateral cerebellar hemispheres (particularly crus 1 and 2) are involved in the executive network and play an important role in executive function [52], [53], and lesions of the lateral cerebellar hemispheres may affect smooth pursuit eye movement [54]. Previous studies have also provided evidence that the posterior lobe of the cerebellum is more closely associated with cognitive roles in motor learning, hand-eye coordination, error detection and correction, and the control of motor timing [55], [56], [57]. The roles of the oculomotor vermis (lobuli 6 and 7) in these functions are especially well documented [58]. Furthermore, recent findings suggest that visuo-motor coordination is decreased in amblyopia subjects. For example, Grant et al. [59] studied the prehension skills of subjects with amblyopia and found that patients exhibited spatio-temporal deficits in the final approach phase of reaching and grasping. Suttle et al. [60] found amblyopic children spent almost twice as much time grasping objects and made many more errors (1.5–3 times) during reach direction and grip positioning than normal sighted controls. Niechwiej-Szwedo et al. [61] found amblyopia affected both the programming and the execution of visually guided reaching. Our finding of decreased spontaneous brain activity in the precuneus (part of BA7) and the cerebellum (cerebellum area 1–2, 6) further support the role of neurophysiological deficits of visuo-motor integration processing in amblyopia.

The areas with decreased ReHo indices in the prefrontal cortices (part of BA10, 11, 45, 47) correspond partly with Broca's area and the orbitofrontal cortex. Broca's area is the region devoted to speech production and also plays a role in various cognitive and perceptual tasks [62]. The orbitofrontal cortex, which includes the medial portions of BA 9, 10, 11, and the inferior portions of BA 45, 47 in the human brain [63], is considered part of the limbic system and is involved in sensory integration and reward expectation [64]. These decreased ReHo indices may reflect decreased integration abilities in amblyopia.

Thus, we assumed that the decreases in ReHo in these regions might represent a new measure of decreases in fine visuo-motor integration processing ability in anisometropic amblyopia.

### Increased ReHo of spontaneous brain activity in amblyopia

Notably, the amblyopia subjects showed increased ReHo indices in the PostCG, PreCG, PCL, and STG (BA41); these areas correspond to the primary somatosensory cortex, primary motor cortex, motor area, and primary auditory cortex, respectively. Additionally, the insula is thought to be a non-primary motor area, and it plays roles in the regulation of the body's homeostasis, motor control, self-awareness, cognitive functioning, and interpersonal experience [65]. It is generally believed that the visual areas are functionally connected to the somatosensory area [66], [67], [68], [69], [70], [71], motor area [71], [72], [73], and auditory area [71], [74]. Our finding of increased ReHo of spontaneous brain activity in these areas may reflect plasticity that compensates for amblyopia-related deficits. This potential compensatory mechanism has also been suggested in subjects with visual deficits such as blindness [71], [73], [75], [76] and neuromyelitis optica [32], [77], and these mechanisms may be general changes that enable visually impaired subjects to perform sensory-guided motor behaviors.

Increases in ReHo were also found in the parts of BA 18 and BA 19 that correspond to visual area V2, and visual area V3 (Dorsal V3 and Ventral V3) (Figure 1, Table 3). This finding is consistent with a previous, related study of the blind [33]. V2 receives strong feed-forward connections from V1 (direct and indirect via the pulvinar) and sends strong connections to V3, V4, and V5. V2 also sends strong feedback connections to V1. Dorsal V3 receives inputs from V2 and the primary visual area and projects to the posterior parietal cortex. Ventral V3 has much weaker inputs from the primary visual area and stronger connections with the inferior temporal cortex. The increased ReHo in the visual system may reflect compensatory plasticity in the feed forward system from the primary visual cortex and/or weakened feedback from the higher extra-striate visual cortex in the visual pathway. We also found increased ReHo in the left fusiform gyrus, which is consistent with some scattered reports of cortical adaptations in adults with amblyopia [78].

### Limitations and Caveats

Interestingly, we did not find significant alterations of spontaneous activity in the visual cortices of amblyopia patients at the current statistical threshold. However, when we employed a relatively loose threshold, we found that the ReHo indices of visual brain regions were altered in amblyopia (Figure S2). We assume this may be because we measured spontaneous activity patterns, while previous studies mainly focused on activation patterns in response to visual stimuli in amblyopia subjects. Another possible reason might be that spontaneous activities are stronger in motor areas, and these effects overshadow any effects within the visual cortex. Further, the relatively small sample of this study may have prevented us from finding positive results in the visual cortex in the amblyopia subjects.

Additionally, in our study, we did not find statistically significance differences between the amblyopia subjects and subjects with normal vision in terms of gray matter volume with the VBM method. Furthermore we did not find significantly altered gray matter volumes in the identified ReHo regions. However, it is hard to conclude that morphologic changes do not occur in the cortex of humans with amblyopia.

We also found that the ReHo measure correlated with disease severity in the patient group (Figure 2B). However, this correlation must be considered preliminary due to the small sample size. Furthermore, our results should be interpreted carefully because we did not classify our patients based on left or right eye impairments due to the small sample size. In the future, an investigation with a large sample is required to reduce individual effects on the results.

## Supporting Information

Figure S1 Plot of the mean gray matter volumes in which ReHo values were not significantly different between the two groups.
(TIF)

Figure S2 Brain areas with altered ReHo in the anisometropic amblyopia individuals (*P<0.05*, 30 voxels). (Red indicates ReHo indices that were higher in subjects with normal vision, and blue indicates ReHo indices that were higher in subjects with anisometropic amblyopia individuals).
(TIF)
