# Supplementary figures and images for: Altered Spontaneous Activity in Anisometropic Amblyopia Subjects: Revealed by Resting-State fMRI
Source: PLoS One. 2012 Aug 24;7(8):e43373. doi: 10.1371/journal.pone.0043373 (PMC3427333; doi:10.1371/journal.pone.0043373)

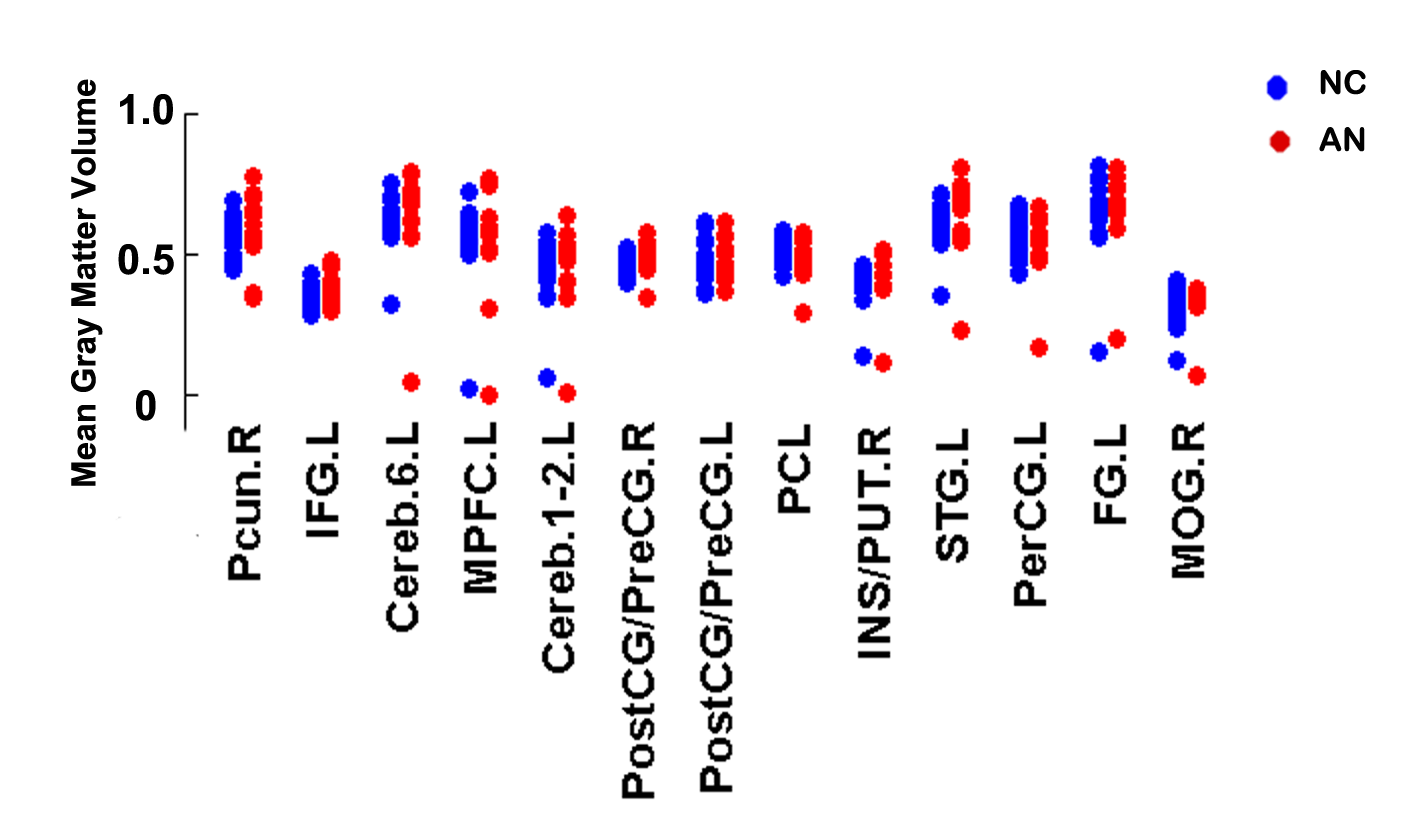

Supplement: Figure S1 — Plot of the mean gray matter volumes in which ReHo values were not significantly different between the two groups. (TIF) [file pone.0043373.s001.tif]

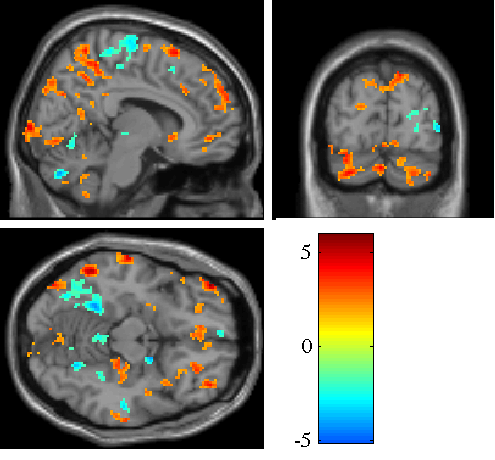

Supplement: Figure S2 — Brain areas with altered ReHo in the anisometropic amblyopia individuals (P<0.05, 30 voxels). (Red indicates ReHo indices that were higher in subjects with normal vision, and blue indicates ReHo indices that were higher in subjects with anisometropic amblyopia individuals). (TIF) [file pone.0043373.s002.tif]
